# Modeling Epac1 interactions with the allosteric inhibitor AM-001 by co-solvent molecular dynamics

**DOI:** 10.1007/s10822-020-00332-y

**Published:** 2020-07-22

**Authors:** Marianna Bufano, Marion Laudette, Jean-Paul Blondeau, Frank Lezoualc’h, Marianna Nalli, Romano Silvestri, Andrea Brancale, Antonio Coluccia

**Affiliations:** 1 Department of Drug Chemistry and Technologies, Istituto Pasteur Italia – Fondazione Cenci Bolognetti, Sapienza University of Rome, Piazzale Aldo Moro 5, 00185 Rome, Italy; 2 Institut des Maladies Métaboliques et Cardiovasculaires, INSERM UMR-1048, Cedex 04, 31432 Toulouse, France; 3 Université de Toulouse - Paul Sabatier, Cedex 04, 31432 Toulouse, France; 4 Faculté de Pharmacie, Université Paris-Sud, Châtenay-Malabry Cedex, 92296 Paris, France; 5 Cardiff School of Pharmacy and Pharmaceutical Sciences, King Edward VII Avenue, Cardiff, CF103NB UK

**Keywords:** EPAC, Molecular Dynamics, Cosolvent Molecular Dynamics, Docking, PCA

## Abstract

The exchange proteins activated by cAMP (EPAC) are implicated in a large variety of physiological processes and they are considered as promising targets for a wide range of therapeutic applications. Several recent reports provided evidence for the therapeutic effectiveness of the inhibiting EPAC1 activity cardiac diseases. In that context, we recently characterized a selective EPAC1 antagonist named AM-001. This compound was featured by a non-competitive mechanism of action but the localization of its allosteric site to EPAC1 structure has yet to be investigated. Therefore, we performed cosolvent molecular dynamics with the aim to identify a suitable allosteric binding site. Then, the docking and molecular dynamics were used to determine the binding of the AM-001 to the regions highlighted by cosolvent molecular dynamics for EPAC1. These analyses led us to the identification of a suitable allosteric AM-001 binding pocket at EPAC1. As a model validation, we also evaluated the binding poses of the available AM-001 analogues, with a different biological potency. Finally, the complex EPAC1 with AM-001 bound at the putative allosteric site was further refined by molecular dynamics. The principal component analysis led us to identify the protein motion that resulted in an inactive like conformation upon the allosteric inhibitor binding.

**Electronic supplementary material:**

The online version of this article (10.1007/s10822-020-00332-y) contains supplementary material, which is available to authorized users.

## Introduction

The cyclic adenosine monophosphate (cAMP) is a universal second messenger that regulates many biological processes, including cell proliferation, differentiation, and apoptosis [1]. The effects of cAMP in mammalian cells are mediated by at least three effector families: protein kinase A (PKA), exchange proteins activated by cAMP (EPAC) and ion channels bearing a cyclic nucleotide binding domains (CNBD). The EPAC proteins regulate a variety of physiological processes, such as calcium homeostasis in cardiomyocytes, insulin secretion from pancreatic β cells, integrin-mediated cell adhesion, and cell death [2]. Therefore, EPAC proteins are considered as targets for a wide range of therapeutic applications [3]. To date, two EPAC isoforms have been identified, EPAC1 and EPAC2 which display distinct pattern of tissue expression. Indeed, EPAC1 is ubiquitously expressed whereas EPAC2 and its slice variants are localized in the brain (EPAC2A), pancreatic cells (EPAC2B) testis and liver (EPAC2C) [4].

**Fig. 1:**
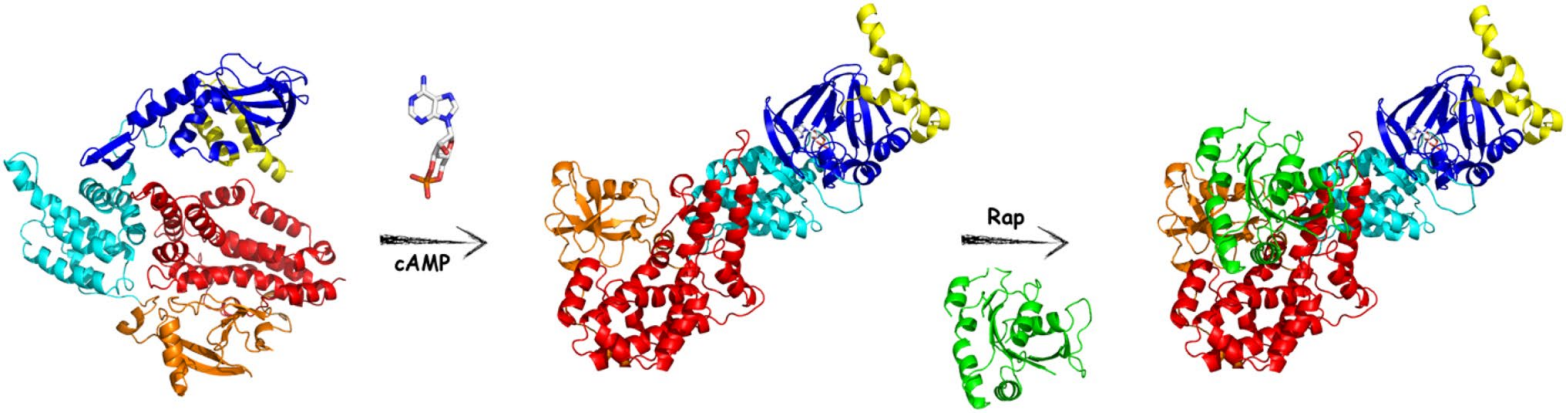
Graphical representation of EPAC activation upon cAMP binding. CNBD, DEP, REM, RA, and CDC25-HD domains are coloured in yellow, blue, cyan, orange and red respectively. cAMP is reported as white stick, RAP is reported as green cartoon. The cyclic nucleotide binding allows the regulatory domain to open leading the catalytic region exposed for binding of Rap

EPAC1 and EPAC2 are multidomain proteins featured by an N-terminal regulatory region (RR) and a C-terminal catalytic region (CR). The amino-terminal regulation region contains a Disheveled/Egl-10/pleckstrin (DEP) domain followed by a cyclic nucleotide-binding domain (CNBD). EPAC2A has an additional low-affinity CNBD, which is unable to induce guanine nucleotide exchange factor (GEF) activity after cAMP binding [5]. The catalytic region consists of a Ras exchange motif (REM), a Ras association domain (RA) and a CDC25 homology domain (CDC25HD). The CDC25HD catalyses GDP-GTP exchange for Rap, while the REM domain contributes to stabilize a catalytic helix, and the RA domain influences EPAC subcellular localization [6]. The regulatory CNBD at the RR C-terminus [7, 8] controls the relative orientations of the RR and CR with respect to each other. In the absence of cAMP, the two regions adopt a closed conformation, in which the RR restricts access of the Rap GTPases to the CR, resulting in constitutive inhibition. Upon binding of cAMP to the conserved CNBD, the two regions adopt an open (active) topology, where the Rap substrate can interact with the CR to promote GDP-GTP exchange [7–10] (Fig. 1).

In the heart, cAMP represents one of the most important mechanism for increasing cardiac function in response to acute sympathetic stimulation of β-adrenergic receptor (β -AR) [11]. Although acute activation of b-AR has beneficial effect on cardiac function, chronic b-AR stimulation of this pathway promotes pathological cardiac remodelling, which may ultimately lead to heart failure (HF) [12]. Importantly, recent data show pharmacological or genetic inhibition of EPAC1 prevents cardiac hypertrophy and fibrosis induced by sustained β-AR activation and improves cardiac function [13].

Therefore, targeting EPAC1 may have potential therapeutic benefits in cardiac diseases [13, 14] (Fig. 1).

Currently, there are only few EPAC1 modulators [14]. Based on their chemical structure these compounds may be divided in cAMP analogue and non-nucleotidic small molecules. The major drawbacks about the reported modulators were the lack of selectivity between the EPAC isoforms or PKA, and the unsuitable drug-like properties. Another concern about modulator of cAMP-regulated protein is that the orthosteric cAMP site is amply conserved making selectivity a crucial issue [15]. This task may be solved looking for non-competitive allosteric modulator that is expected to have reduced risks of cross-reactivity with other receptors that signal via cAMP such as PKA or CNG ion channels [16]. To our knowledge there are just two compounds, CE3F4 [17, 18] and AM-001 [13], that are able to selectively inhibit EPAC1. The small molecule AM-001 is a thieno[2,3-b]pyridine derivative (Table S1) acting as a selective non-competitive antagonist of EPAC1 [13]. AM-001 mitigates cardiac hypertrophy, inflammation and fibrosis, and improves cardiac function during chronic β-adrenergic receptor activation. In in-vivo experiments, AM-001 reduces the infarct size after mouse myocardial ischaemia/reperfusion injury [13].

Since the biological evaluation suggested a non-competitive mechanism of action for the AM-001 derivative [13] we sought to identify its putative allosteric binding site. To date, there are not experimental data about the allosteric site localization. It was just reported that the hinge region of EPAC might be a druggable region because of its crucial role in the close to open conformation transition [19, 20].

The cosolvent molecular dynamics (CMD) method is very useful to highlight both catalytic and allosteric sites [21]. This method was inspired by the multiple solvent crystal structures (MSCS) technique [22]. The MSCS is based on solving crystal structures of proteins in the presence of various organics cosolvents. Overlapping locations of different cosolvents were found to be highly correlated with regions of biological significance [21]. The CMD could be considered the in-silico counterpart of the MSCS experiments. The CMD method uses the molecular dynamics simulation of a protein solvated by an organic solvents/water mixtures to determine the sites where the organics solvents preferentially bind.

In our work, we attempted to identify the EPAC1 allosteric binding site by using CMD. Thus, the EPAC1 active and inactive conformations were solvated with a solution of 20% (w/w) of isopropanol (ISO), ethanol (ETOH), and dimethylsulfoxide (DMSO), in water, then the cosolvent occupancy maps were generated and investigated.

ISO is the most used solvent for CMD as it is able to interact with hydrophobic protein sites [23]. DMSO and ETOH are chosen for their hydrogen-bond acceptor and donor capabilities respectively [24, 25]. The cosolvents were also chosen to be fully water miscible to avoid molecules aggregations phenomena and the concentration was fixed to avoid protein denaturation [26].

The models of the inactive and active EPAC1 conformations were generated by homology from the EPAC2 crystal structure (inactive state: PDB code 2BYV [27]; active state PDB code 3CF6 [6]).

To evaluate that the unfolding phenomena did not affect the protein during the simulations the RMSD of the backbone Cα was computed [28]. The RMSD values showed that cosolvents molecules did not disturb the protein folding (Figs. S1, S2).

**Fig. 2:**
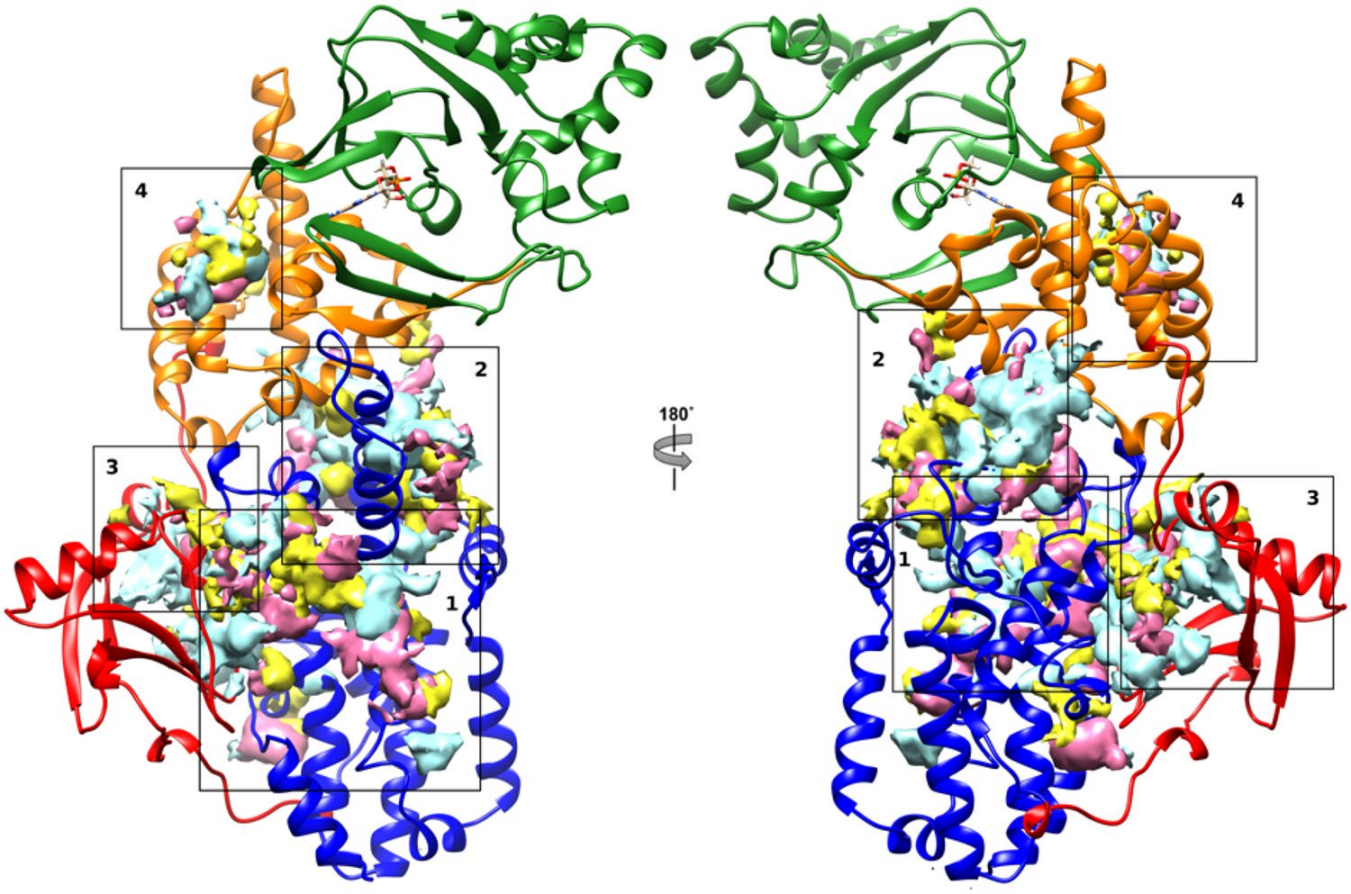
Epac1 active conformation cosolvent occupancy maps selected areas. Epac1 is reported as cartoon: CNBD and DEP green; REM orange; CDC25-HD blue and RA red. Yellow maps are for ETA; cyan for ISO and pink for DMSO

**Fig. 3:**
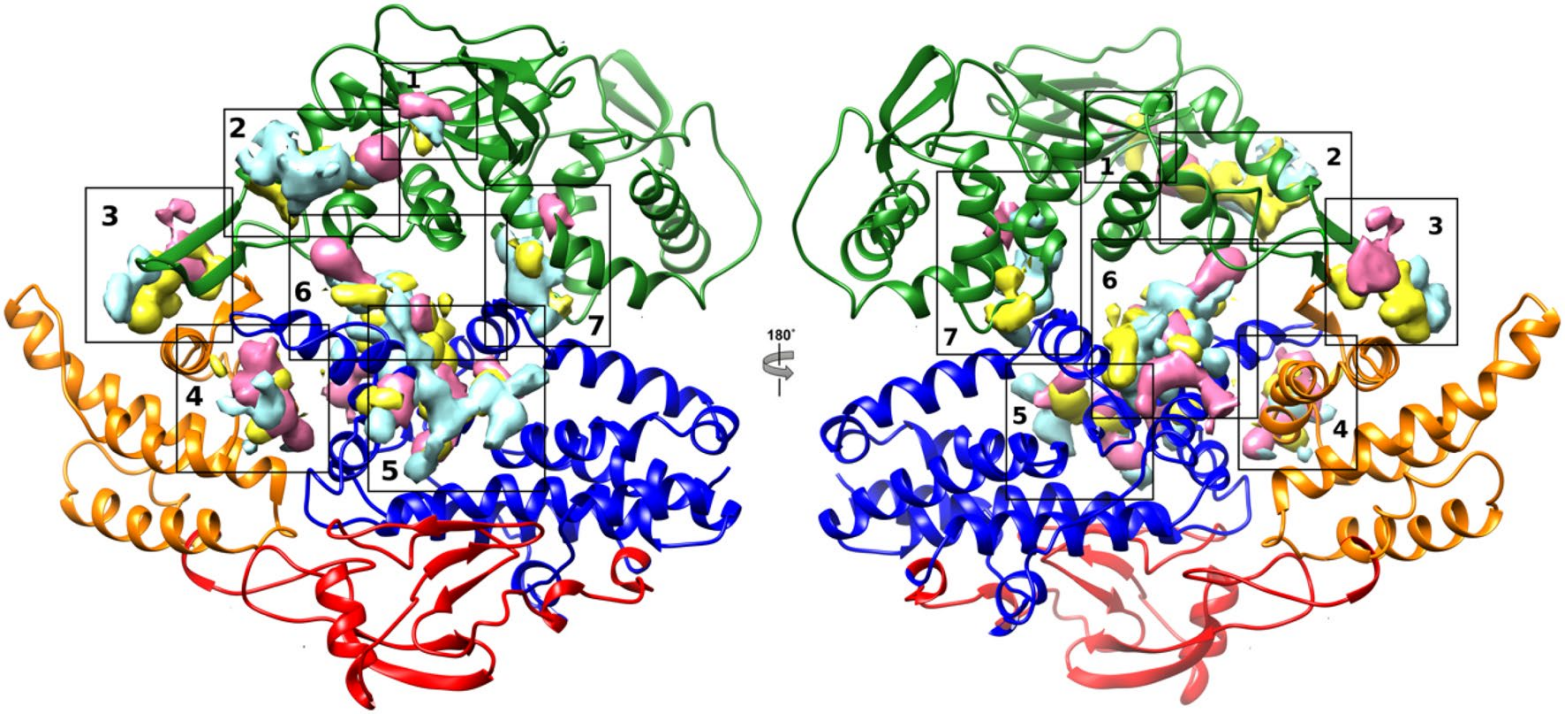
Epac1 inactive conformation cosolvent occupancy maps selected zones. Epac1 inactive conformation is reported as cartoon: CNBD and DEP green; REM orange; CDC25-HD blue and RA red. Yellow maps are for ETA; cyan for ISO and pink for DMSO

The trajectory of each simulation was analysed to determine the cosolvent occupancy maps. Each map indicates the pockets where the cosolvent molecules were most frequently located. The interaction energy among protein and cosolvent molecules was not taken into account. A size cut-off was applied to remove the smaller disconnected part of the maps doing interpretation easier. Thus, the protein areas where the cosolvent occupancy maps were superimposable, represented the suitable sites for a partner binding (Figs. 2 and 3 and S3, S4).

## Materials and methods

### EPAC structures

The EPAC structure was downloaded from the PDB data bank (http://www.rcsb.org/), inactive state: PDB code 2BYV; [27] active state PDB code 3CF6 [6].

Homology model were carried out by homology model PRIME [29] of MAESTRO [30]. The obtained models were examined to avoid steric clashes, bond length deviations and angles, etc., by using the Ramachandran plot and the Protein Preparation Wizard. [31] The missing loops were obtained by Raptor X [32, 33]. The primary sequence of EPAC1 and EPAC2 was downloaded by UniProtKB (https://www.uniprot.org) code: O95398NCBI and UniProtKB code: Q8WZA2 respectively.

## Molecular dynamics

Molecular dynamics was performed with the AMBER 12 suite [34]. For simulation in water the protein was solvated in a periodic octahedron simulation box using TIP3P water molecules, providing a minimum of 10 Å of water between the protein surface and any periodic box edge. The organic cosolvent/water box were prepared by packmol by fixing a 20% w/w ratio [35]. Parameters for cosolvents were obtained by pyMDMix. (http://mdmix.sourceforge.net) Then, ions were added to neutralize the charge of the total system. The solvents molecules and the ions were energy-minimized keeping the coordinates of the protein-ligand complex fixed (1000 cycle), and then the whole system was minimized (5000 cycle). Following minimization, the entire system was heated to 298 K (20 ps). The production simulation was conducted at 298 K with constant pressure and periodic boundary condition. Shake bond length condition was used (ntc = 2). Production was carried out on GeForce gtx780 gpu. The cosolvent occupancy maps were computed with trajectories length of 100 ns and 250 ns. The obtained maps were similar regardless the length of the trajectories.

The production length of the simulations to evaluate the AM-001 stability at the studied sites was 10 ns. The production time to run PCA was 200 ns. All simulations were repeated five times. The occupancy maps were calculated by Chimera Volume viewer [36]. Chimera we also used for the representation of the maps by volume viewer range 50%, and for the size filtering by the Hide Dust tool size 5.0 [37]. Compounds were parametrized by Antechamber [38, 39] using BCC charges. Trajectories analysis were carried out by cpptraj program [40].

### PCA

The PCA analyses were carried out by cpptraj program [40]. The trajectory was firstly superimposed to an average structure obtained by cpptraj. Then, the trajectory of the a-carbon was extracted and the covariance matrix calculated. The diagonalization of the covariance matrix generated a set of eigenvectors that gave a vectorial description of the motion. The computed eigenvectors and eigenvalues were used to draw the porcupine plot by means of the Normal Mode Wizard module (NMWiz) [41, 42] of the VMD gui [43].

## Molecular docking

Ligand structures were built with MAESTRO [30] and minimized using the OPLS3 force field until a rmsd gradient of 0.05 kcal/(mol Å) was reached. The docking simulations were performed using Gold, [44] Plants [45] and Autodock [46]. We set a binding lattice of radius or side large enough to cover the whole studied maps, then all default settings were used. The Docking of the AM-001 analogues were carried out by Plants [45], using as receptor a representative structure obtained by the trajectory of the EPAC1/posa3_6. The representative structure was the average structure extracted by cpptraj [40] after a stable rmsd was reached. The poses binding free energy was calculated by MAESTRO prime MMGB-SA module [47]. The pictures reported in the manuscript were done with Pymol [48].

## Results and discussion

### Analyses of active conformation occupancy maps

The active conformation of the enzyme was mainly studied to access the quality of the method. The inhibition of EPAC1 by AM-001 was previously determined by BRET assays [17]. Indeed, the experiment measured the level of activation of the EPAC1, namely the shift from inactive to active form of the enzyme after the antagonist binding. The cosolvent occupancy maps were computed with trajectories of 100 ns (Fig. S3) and 250 ns (data not shown). The occupancy maps analyses for the EPAC1 active conformation led to the identification of four interesting zones signed as areas from 1 to 4 (Fig. 2) regardless of the trajectories length.

The areas 1 and 2 were located at the CDC25-HD domain and corresponded to the Rap binding site [6]. Indeed, the superimposition of the EPAC2 crystal structure (PDB code: 4MGI) [49], bearing Rap interacting domain, with the EPAC1 model clearly showed that the areas 1 and 2 were filled by this domain. Specifically, we observed that the area 1 involved 4 α helices of the CDC25-HD namely α1 (671–680), α3 (708–713), α5 (750–756) and α9 (839–844). This area bound the β4 and the loop linking β4 to α3 of the Rap domain. (Fig. S5) The area 2 involved the CDC25 Helical Hairpin (α9 and α10 from 823 to 854) that accommodated the loop between α1 and β2 of Rap [50]. (Fig. S5) The area 2 was also reported to be potentially involved in an interaction between EPAC1 and RanPB2 [51].

The site 3 was located at the interface between the CDC25-HD and RA domains, and comprised a loop absent in EPAC2. This broad surface encompassed part of the loop (550–557), the CDC25-HD α1 (678–682) and the β1 (558–562) of RA domain. This area was reported to be suitable for a protein-protein interaction [52].

The area 4 was located at the REM domain. It comprised the α1 (394–398) and α3 (434–440) helices of REM and a small loop of CNBD (294–299). This zone was identified to be suitable for partners binding by FTPmap experiments [20].

Various proteins with undefined binding pockets, contributed to the regulation and/or the subcellular localization of EPAC1 [51]. Indeed, it was reported that the residues 838–881 were involved in the compartmentalization of EPAC1, but it was not clarified how these residues drove this activity [52]. Furthermore, tubulin was also reported as a direct partner of EPAC1, but its binding site was not yet identified [53]. Our analyses of the occupancy maps suggested that some areas were suitable for binding of partner proteins are consistent with the available structural data, validating the appropriateness of the method used.

### Occupancy maps analyses of inactive conformation

The CMD procedure was then applied to the EPAC1 auto-inhibited form (inactive conformation) with the aim to identify a putative binding site for AM-001. The cosolvent occupancy maps were computed by trajectories of 100 ns (Fig. 3) and 250 ns (Fig. S6). The analyses of the cosolvent occupancy maps did not show any significant differences depending on the trajectories length and led to the identification of some attractive areas. The proposed mechanism of cAMP-induced EPAC1 activation involves EPAC1 regulatory domain that moves away from the catalytic domain. In this transition a crucial role is played by the lid (first β-strand of REM 384–391 and the tip of CDC25-HD HP 832–837) and the hinge helix (CNBD 299–308). Accordingly, AM-001 binding to its allosteric pocket should have to impair this cAMP-dependent conformational change [20]. These data were used to reject the area located at the CDC25-HD and the RA domains. Indeed, the areas appear to be too far from both the lid and hinge helix to influence the inactive to active conformation state. This observation led us to managed 7 areas (Fig. 3).

Area 1 was the cAMP binding pocket [6]. The identification of the cAMP site further validated the reliability of the method.

The remaining areas were inspected by docking and molecular dynamics. Area 2 was located at the CNBD close to the catalytic site and it encompassed CNDB α3 and the following loop (245−250), α4 (251–260) and α6 (344–350). Area 3 was at the interface between CNBD and REM domains. This site is delimited by REM β1 (387–392) and α1 (398−402) together with the CNBD β9 (353–359) and β10 (363−368). Area 4 involved the HP loop of CDC25-HD and the REM α2 (423−430). Area 5, was the only one entirely located at the CDC25-HD comprising the α2 (689−692), α3 (704−708), α5 (761−765) and α9 (815−827). The area 6 was at the interface of CDCD25-HD and REM. The area was defined by the α8 (800–805), α9 (817–822) and α10 (844–851) of CDC25-HD and the loop between α2 and α3 (247–251) of the CNDB. Lastly, area 7 was located between the CDC25-HD and the CNDB domains involving the α8 (793–801) of the CDC25-HD and the long helix (206–216) linking CNDB to the Dep domain. (Fig. S7)

Thus, AM-001 was docked to each of the listed areas. The docking studies were carried out with Gold, [44] Plants [45] and Autodock [46].

For each area, at least one consistent AM-001 binding pose (RMSD < 1.5 Å) among the docking software was selected and submitted to molecular dynamic simulations (10 ns).

For the areas 2, 3, 4 and 7 the analyses of the molecular dynamic trajectories showed that the ligand moved out of the pocket at the early stage of the simulation. In fact, these sites were markedly solvent exposed. This feature was not common for small molecules binding site [54, 55], and it was therefore hypothesized that these sites were more appropriate as protein-protein interaction pocket [56, 57].

Then we managed the two remaining areas, namely 5 and 6. Once again, the selected binding poses were submitted to molecular dynamics simulations. The trajectories analysis showed two stable (rmsf < 1.0 Å) binding modes for area 5 (termed as pose4_5 and pose9_5) (Figs. S9, S10) and one for area 6 (termed as pose3_6).

Since AM-001 preferentially bound to the EPAC1 isoforms (IC_50EPAC1_ 48.5 µM), and it was ineffective in suppressing EPAC2 activity (IC_50EPAC2_ > 1000 µM) [13] we investigated whether the 3 selected binding mode might be suitable for EPAC2. Thus, we carried out a series of molecular dynamics simulations of the 3 binding modes placed in the corresponding pockets of EPAC2. The trajectories and the binding free energy were calculated [58, 59] and compared with those resulting from the EPAC1 simulations.

The trajectories inspection showed that the poses for area 5 were stable for EPAC2 (pose4_5 rmsf 0.49 Å pose9_5 rmsf 0.39 Å) as observed for EPAC1 (pose4_5 rmsf 0.41 Å pose9_5 rmsf 0.60 Å). The computed binding free energies for EPAC1 and EPAC2 were very similar (EPAC1 ΔG pose4_5 = − 53.9, pose9_5 = − 56.1; EPAC2 ΔG pose4_5 = − 63.3 pose9_5 = − 53.1). The difference was just 3 Kcal/mol for pose9_5, and a difference of 10 Kcal/mol for pose4_5 with the best ΔG value for EPAC2.

These data could indicate that area 5 is not be suitable for binding of AM-001 as a selective allosteric inhibition of EPAC1. This site could however correspond to a region of biological significance for the enzymatic activity of both EPAC isoforms.

The same analysis was repeated also for the area 6 pose (pose3_6). The complex EPAC2/pose3_6 was submitted to molecular dynamic. The trajectory inspection clearly showed a level of instability. The compound moved toward the solvent, showing a rmsd higher than 3 Å and a calculated ΔG of -45.9 kcal/mol. The complex EPAC1/pose3_6 trajectory was inspected in the same way. The AM-001 pose showed a general stability with a rmsd of 1.19 Å and a calculated ΔG of -65.1 kcal/mol, thus with about 20 kcal/mol in favour of the EPAC1 binding.

These data suggested that area 6 might be the most suitable for the EPAC1 allosteric inhibition. To validate our idea, we extended the molecular dynamic simulation to 200 ns and we testes the ability of this binding site to match known SARs profile of the compound analogues (Table S1).

**Fig. 4:**
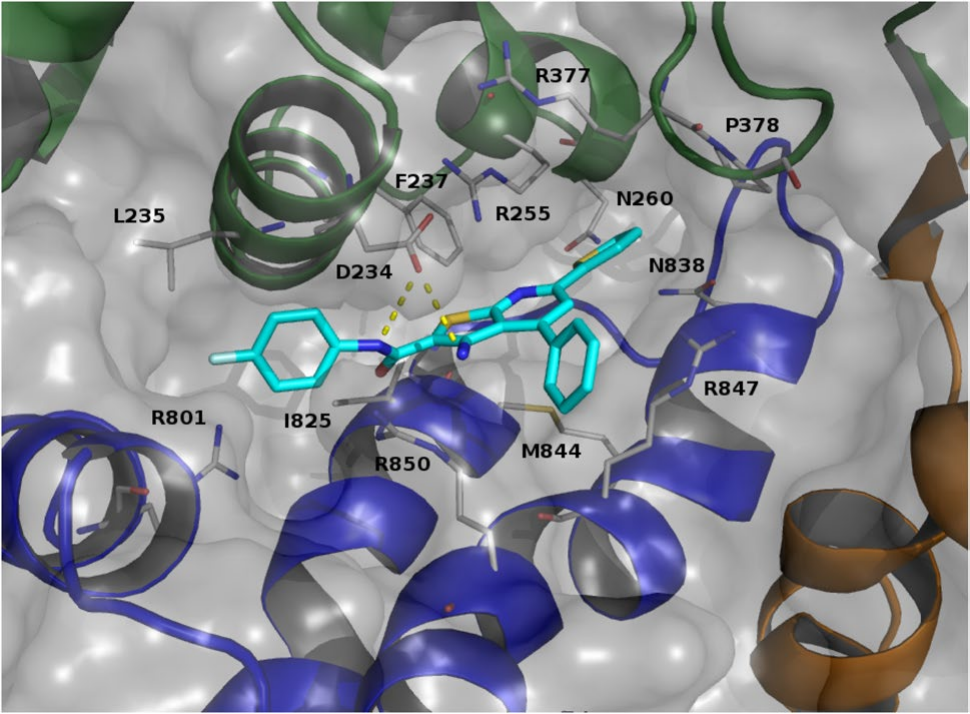
Proposed binding mode for AM-001 (cyan). Epac1 is reported as cartoon: CNBD and DEP green; REMorange; CDC25-HD blue and RA red. Surface is reported in grey. Residues involved in interactions were depictedas grey stick. H-bond was reported as yellow dotted lines

The analyses of the extended trajectory showed that the selected binding mode was stable and led us to identify a series of pharmacophoric interactions: the fluorinated phenyl group had a pi-cation interaction with R801 side chain and hydrophobic contacts with L235 and M; the primary amine moiety as well as the amidic nitrogen atom were involved in H-bond with D234 side chain; the pyridine thiophene fused ring had hydrophobic contacts with F237, R255, I825 and M844 side chains; the unsubstituted phenyl ring was trapped by pi-cation contacts with R847 and R850; the thiophene ring lay in a hydrophobic pocket mainly formed by R377, P378, N260 and N838 side chains; Furthermore, the thiophen aromatic ring behaved as weak H-bond acceptor [60] for the asparagine side chain amide moieties (Fig. 4). These interactions were stable through the simulation time confirming the stability of the proposed binding site.

With the aim to clarify the reason that were responsible for the selectivity of AM-001 between the two studied EPAC isoforms, we compared the two binding sites. The main difference deals with the residues of the loop between the CNBD b10 and REM b1. The loop was 19 residues long for EPAC1 and 18 residues long for EPAC2. Despite the similar length, just two residues were conserved between the two isoforms. Furthermore, the four residues facing the binding pocket for EPAC1(RPPT) were very different from the residues in the same positions of EPAC2 (NQGN). Thus, it may be argued that the behaviour of this loop in terms of conformation and interactions with AM-001 is responsible for the selectivity. Furthermore, R847 and I825, involved in contacts with AM-001 change to N (956) and T (934) respectively in EPAC2, weakening the binding stability. Also, D239 in EPAC1 correspond to E (345) in EPAC2. Taken together these data provide a possible explanation of the AM-001 selectivity toward EPAC1.

Then we docked the nine AM-001 analogues (Table S1) into this binding site, to evaluate the ability of the site to fit the experimental data [13]. The compounds AM-004 and AM-005 which differs just for the fluorine atom position had a very similar binding mode to AM-001, sharing the same interactions. For the compounds AM-003, AM-006, AM-007, AM-008 and AM-009 the binding mode was also comparable to AM-001, albeit with some evident differences: we did not observe the contacts of the fluorinated phenyl ring (AM-003 and AM-006), the thiophene (AM-007 and AM-008), and the unsubstituted phenyl ring (AM-009) of AM-001. For compound AM-002 we obtained a binding mode similar to AM-001, but we did not observe any H-bonds, and the pyrimidone linked phenyl ring was place father from the R801 (Fig. S10). Furthermore, we obtained a good correlation between the computed binding energy for the docking poses [30, 47] and the experimental biological activity with a significant correlation coefficient, R, of 0.74 and Rs of 0.83 p (2-tailed) 0.53% (Table S2). The selected binding mode reasonably fits the SAR for AM-001 analogues. In conclusion, the selected binding mode reasonably fits the published structure-activity relationship for the AM-001 analogues, and seems suitable as a selective allosteric binding site for the inhibitor AM-001.

**Fig. 5:**
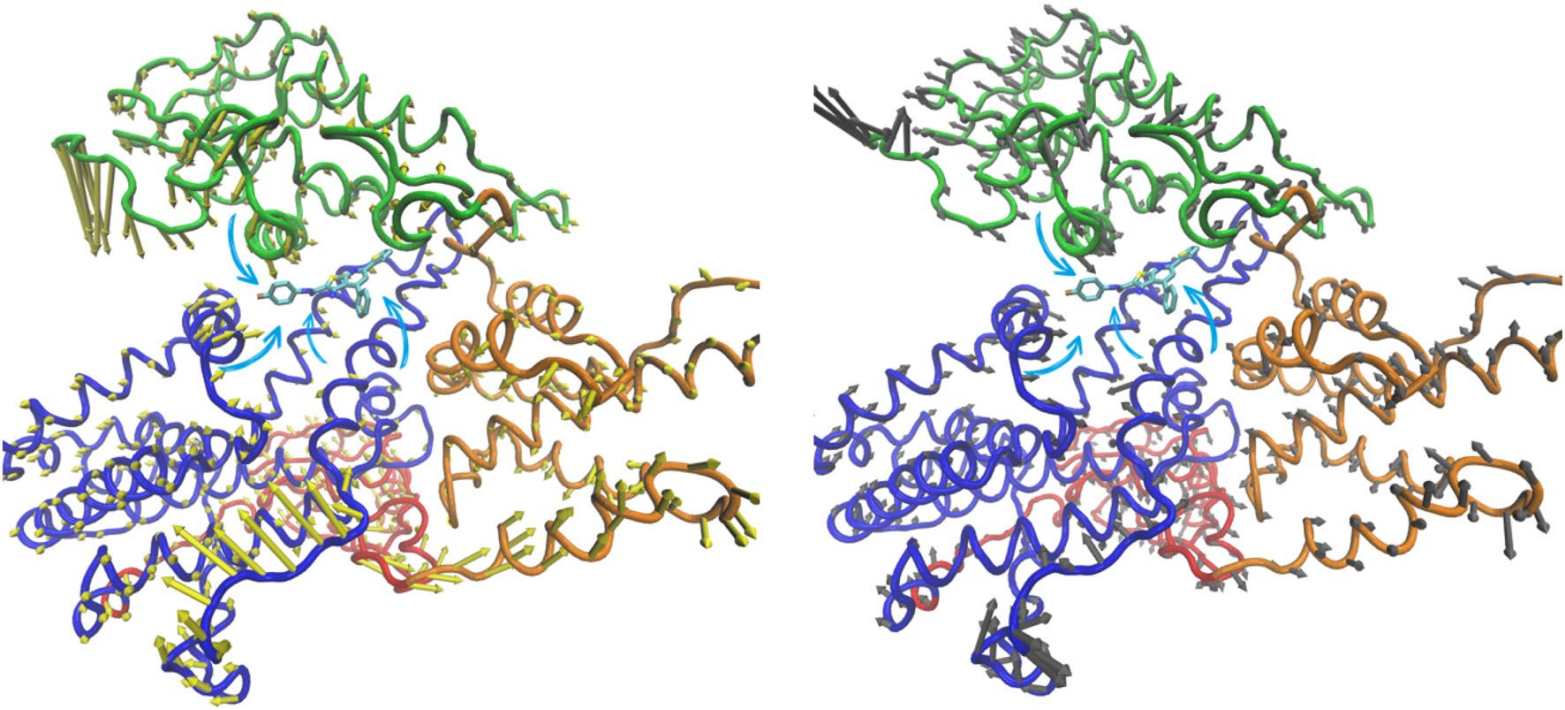
Porcupine plot of the top two eigenvectors. Right panel: eigenvector 1, left panel: eigenvector 2. Epac1 is reported as tube: CNBD and DEP green; REM orange; CDC25-HD blue and RA red. AM-001 is reported as cyan stick. The yellow and grey arrows attached to each a-carbon atom indicate the direction of the movement; the size of each arrow shows the magnitude of the corresponding movement

Lastly, we analysed the EPAC1/AM-001 complex trajectory with the aim of understanding how the AM-001 binding impaired the activating transition of the enzyme. PCA analysis was carried out to highlight the dominants mode of motion of the protein during the simulation time [61–63]. These protein motions were visualized by porcupine plots [64, 65] (Fig. 5) that show the direction and magnitude of the top two eigenvectors (Fig. S12) for each of the backbone Ca atoms. The most prominent observed motion was related to the loops at the CDC25-HD. Focusing on the helices that shaped the binding site we observed that a7 (794–805), a8 (821–830) and a9 (840–850) helices of the CDC25-HD moved toward the CNBD, as well as the CNBD a2 (230–242) moved toward the CDC25-HD.

The observed movements involved the enzyme loop and helices that are crucial in the open -to-close conformational transition. Thus, AM-001 binding to area 6 in the closed conformation of EPAC1 might strengthen the contact between the CDC25-HD and the CNBD domains, stabilizing an inactive-like conformation and preventing the activation transition otherwise induced by cAMP binding to the CNBD domain.

## Conclusions

Cosolvent molecular dynamics were performed with the aim to characterize a putative binding site for the EPAC1 allosteric inhibitor AM-001. By the cosolvent occupancy maps analyses we identified the EPAC1 most suitable sites for a partner binding. Each site was evaluated by the docking and molecular dynamics of AM-001. The pocket located at the interface between a8, a9 and a10 of CDC25-HD and a2 and a3 of CNBD seems suitable as a selective allosteric binding site for the AM-001. The available AM-001 analogues were docked to this site showing a good match with the already reported SAR [13]. The PCA analyses of the EPAC1/AM-001 trajectory highlighted that the AM-001 binding might strengthen the contact between CDC25-HD of the catalytic region and CNBD of the regulatory region stabilizing an inactive “like” conformation. Thus, AM-001 binding to the predicted binding pocket may constrain the highly dynamic EPAC1 in an inhibited conformation, despite the binding of cAMP.

## Electronic Supplementary Material

Below is the link to the electronic supplementary material.

Electronic supplementary material 1 (DOCX 39445 kb)
