# Supplementary material for: Modeling Epac1 interactions with the allosteric inhibitor AM-001 by co-solvent molecular dynamics
Source: J Comput Aided Mol Des. 2020 Jul 22;34(11):1171–9. doi: 10.1007/s10822-020-00332-y (PMC7533256; doi:10.1007/s10822-020-00332-y)
Supplement: Supplementary file 1 — Electronic supplementary material 1 (DOCX 39445 kb) [file 10822_2020_332_MOESM1_ESM.docx]

**Modeling Epac1 Interactions with the allosteric inhibitor AM-001 by co-solvent molecular dynamics**

^1^Marianna Bufano ^2,3^Marion Laudette, ^4^Jean-Paul Blondeau, ^2,3^Frank Lezoualc’h, ^1^Marianna Nalli, ^1^Romano Silvestri, ^5^Andrea Brancale and ^1^Antonio Coluccia

^1^Department of Drug Chemistry and Technologies, Sapienza University of Rome, Laboratory affiliated to Istituto Pasteur Italia – Fondazione Cenci Bolognetti, Piazzale Aldo Moro 5, I-00185 Roma, Italy

^2^INSERM UMR-1048, Institut des Maladies Métaboliques et Cardiovasculaires, 31432 Toulouse, Cedex 04, France;

^3^Université de Toulouse - Paul Sabatier, 31432 Toulouse, Cedex 04, France;

^4^Université Paris-Sud, Faculté de Pharmacie, 92296 Châtenay-Malabry Cedex, France

^5^Cardiff School of Pharmacy and Pharmaceutical Sciences, Cardiff, King Edward VII Avenue, Cardiff, CF103NB, UK

**Supporting information**

Fig. S1 RMSD of Ca atom of Epac1 active conformation simulations Pag. 2

Fig. S2 RMSD of Ca atom of Epac1 inactive conformation simulations Pag. 2

Fig. S3 Cosolvents occupancy maps for Epac1 active conformations. Pag. 3

Fig. S4 Cosolvents occupancy maps for Epac1 inactive conformations 100ns. Pag. 3

Fig. S5 Epac1 active conformation with Rap domain Pag. 4

Fig. S6 Cosolvents occupancy maps for Epac1 inactive conformations 250ns. Pag. 5

Fig. S7 Epac1 sequence and secondary structure. Pag. 6

Fig. S8 EPAC isoforms sequence alignment and residues of areas 5 and 6 Pag. 6

Fig. S9 Epac1 Area5 pose4_5 and pose9_5 binding modes Pag. 7

Fig. S10 Epac2 Area5 pose4_5 and pose9_5 binding modes Pag 7

Table S1 Structures and Bret ratio variation of AM-001 analogues Pag. 8

Figure S11 Plants proposed binding mode of AM-001 analogues. Pag. 9

Table S2 Correlation between BRET-ratio values and calculated ΔG of docking Pag. 10

Figure S12 Graph of the contribution to variance versus eigenvectors number Pag. 11

References Pag. 12

**Fig. S1**


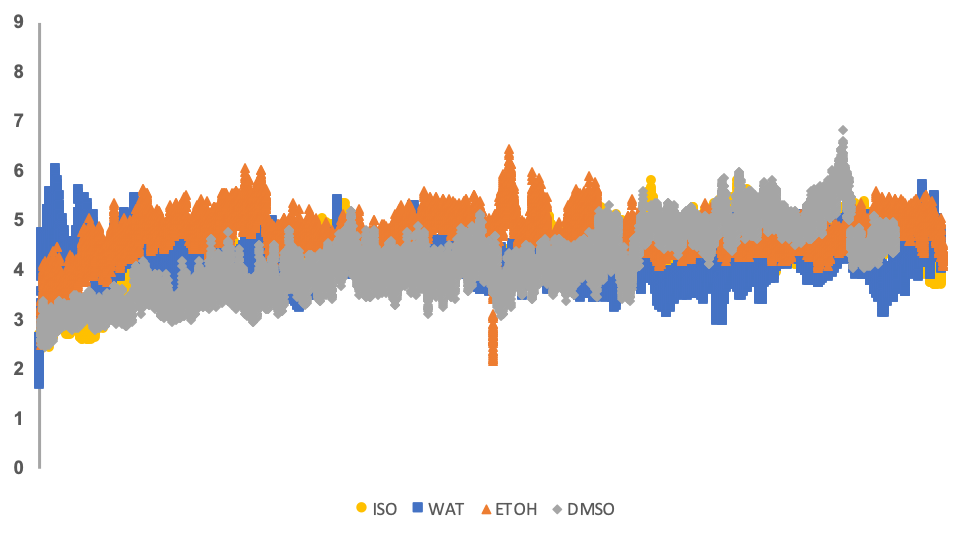


**25000**

**0**

RMSD of Ca atom of Epac1 active conformation simulations. ISO is for isopropanol, Wat is for water, ETOH is for ethanol and DMSO is for dimethyl sulfoxide.

**Fig. S2**

RMSD of Ca atom of Epac1 inactive conformation simulations. ISO is for isopropanol, Wat is for water, ETOH is for ethanol and DMSO is for dimethyl sulfoxide.


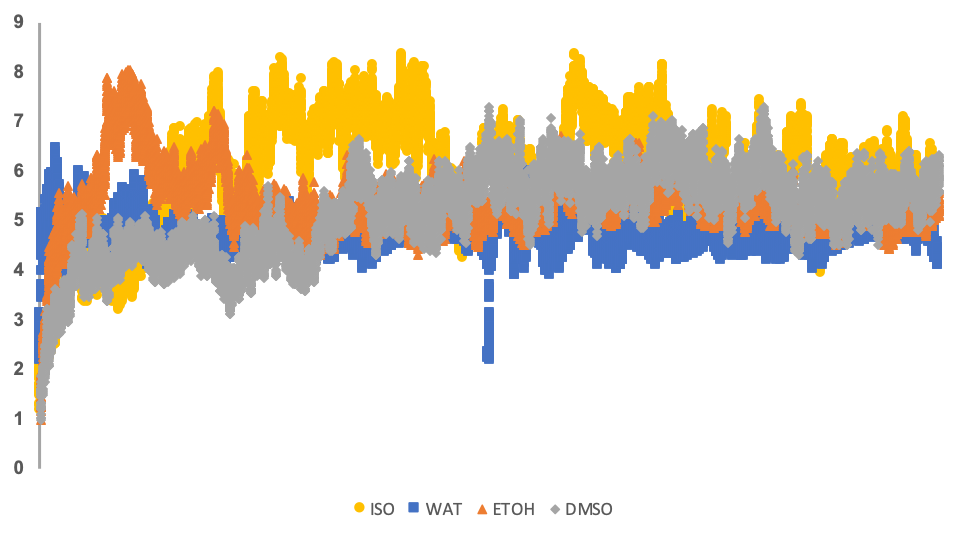


**25000**

**0**

**Fig. S3**

| 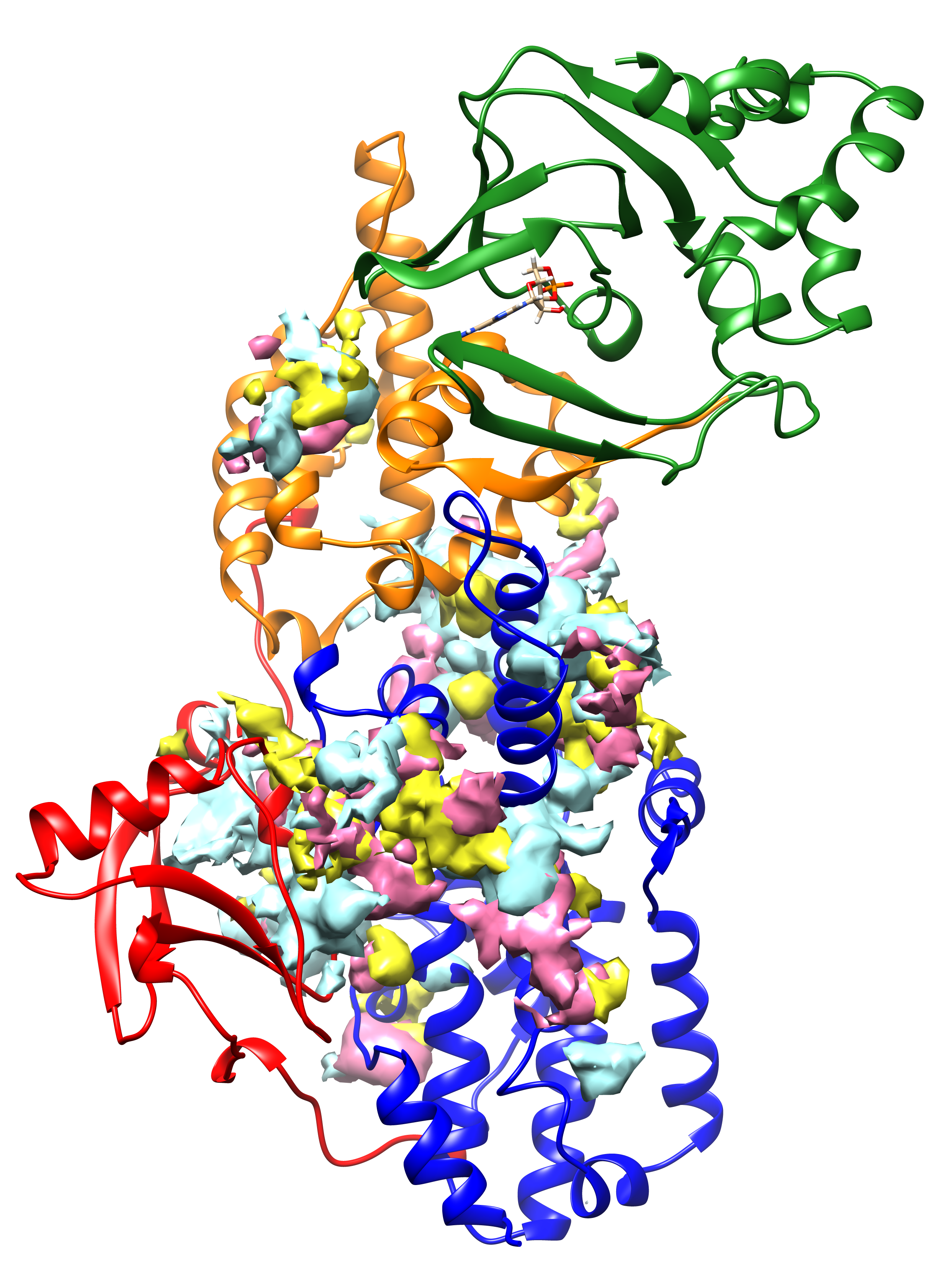 | 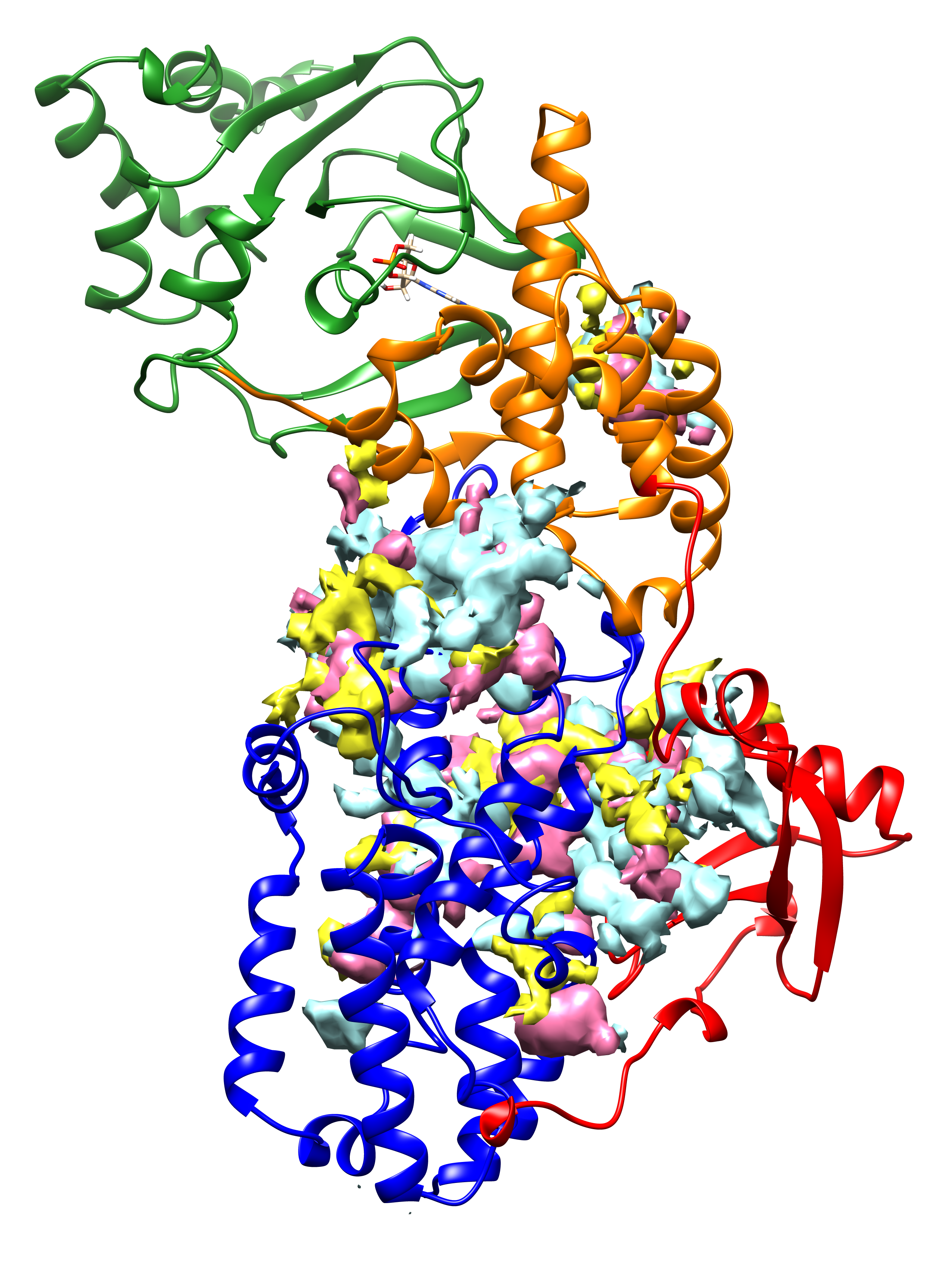 |
| --- | --- |
| Cosolvents occupancy maps for Epac1 active conformations.Epac1 Csolvents occupancy maps for Inactive conformation. Epac1 is reported as cartoon colored by domains: CNBD and DEP green; REM orange; CDC25-HD blue and RA red. Yellow maps are for ETA; cyan for ISO and pink for DMSO. The pictures are rotated of 180 degrees | |

**Fig. S4**

| 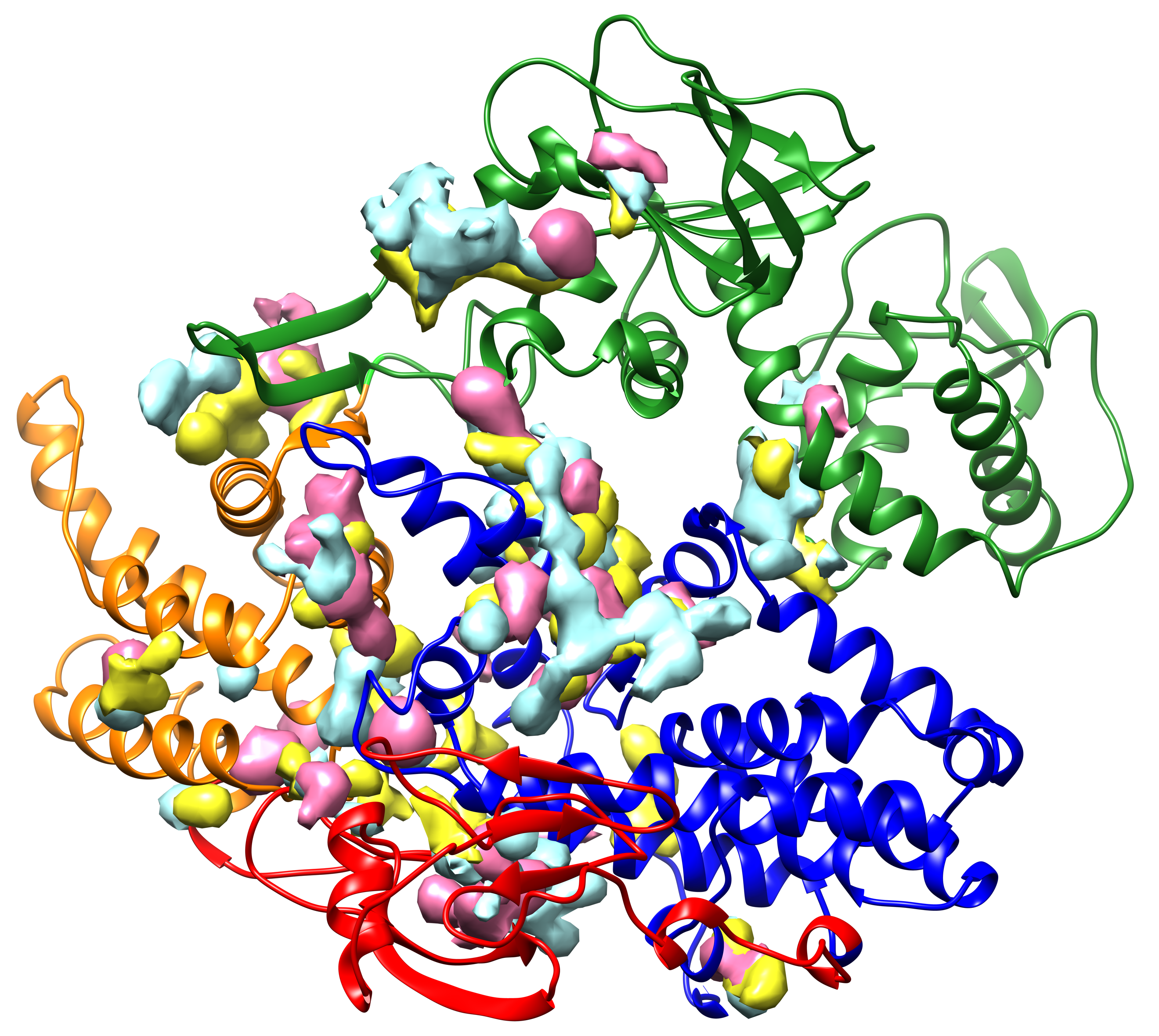 | 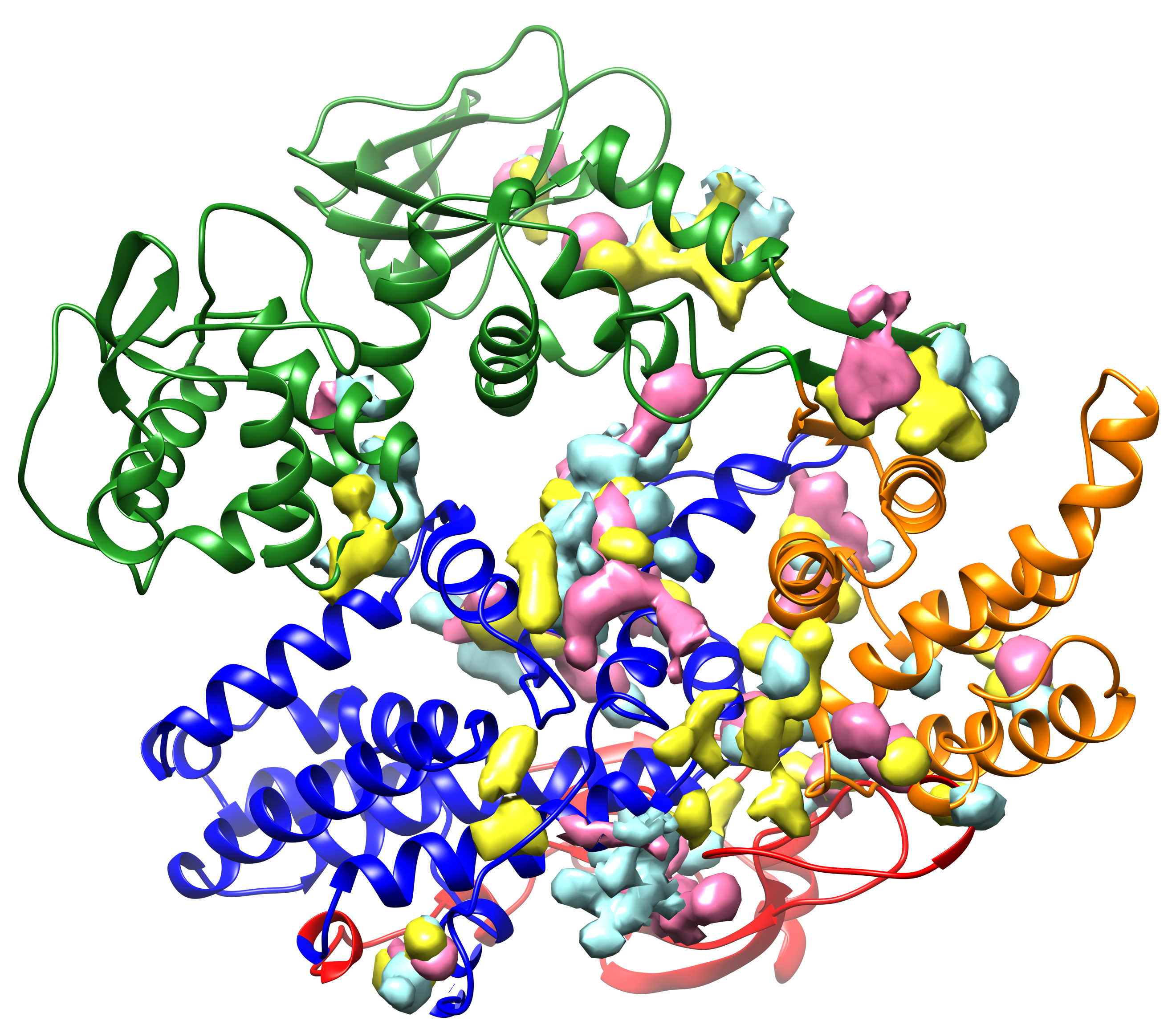 |
| --- | --- |

Epac1 Csolvents occupancy maps for Active conformation. Epac1 is reported as cartoon colored by domains: CNBD and DEP green; REM orange; CDC25-HD blue and RA red. Yellow maps are for ETA; cyan for ISO and pink for DMSO. The pictures are rotated of 180 degrees.

**Fig. S5**

| 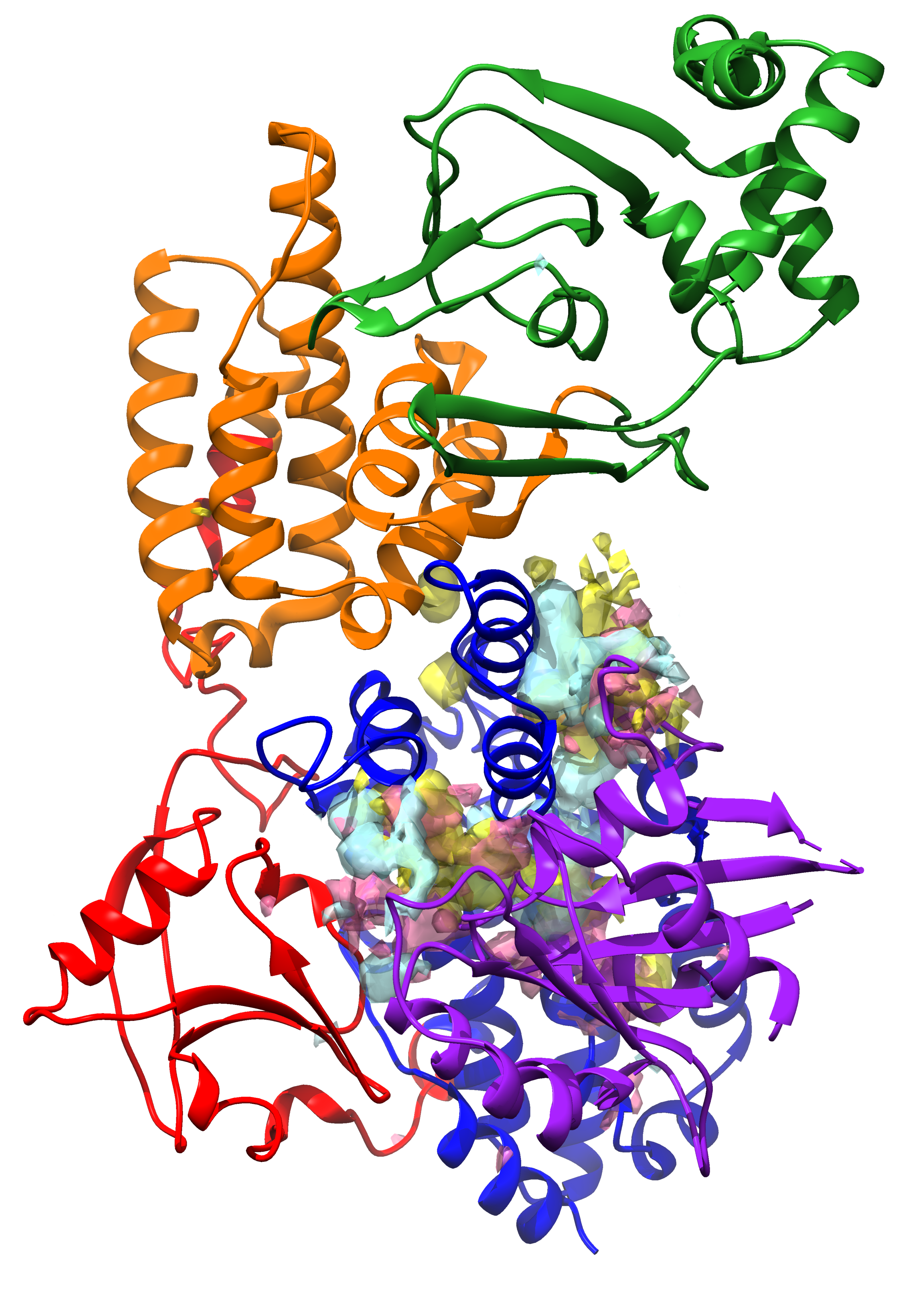  **Area 1**  **Area 2** | 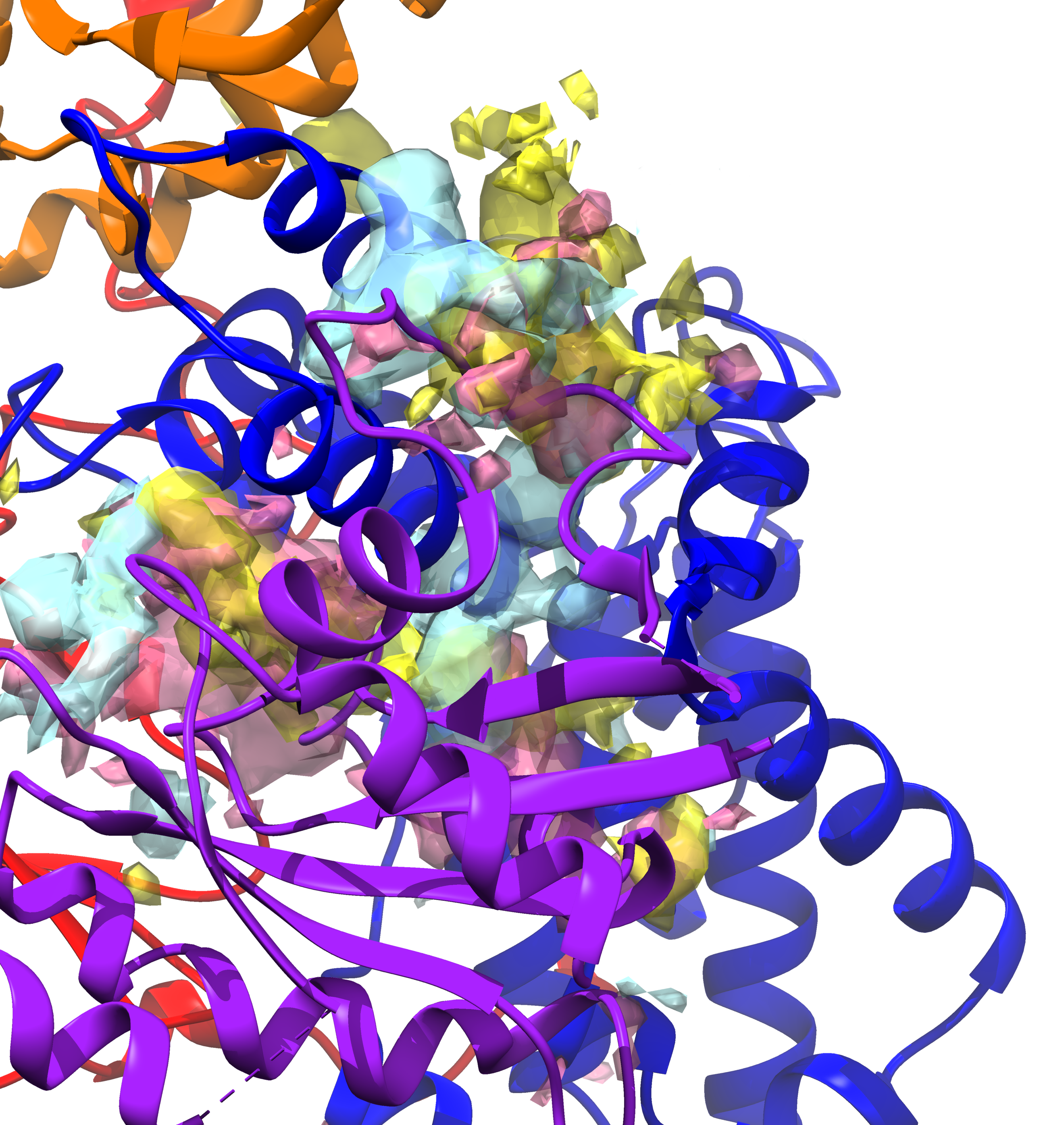  **Area 2** |
| --- | --- |
|  | 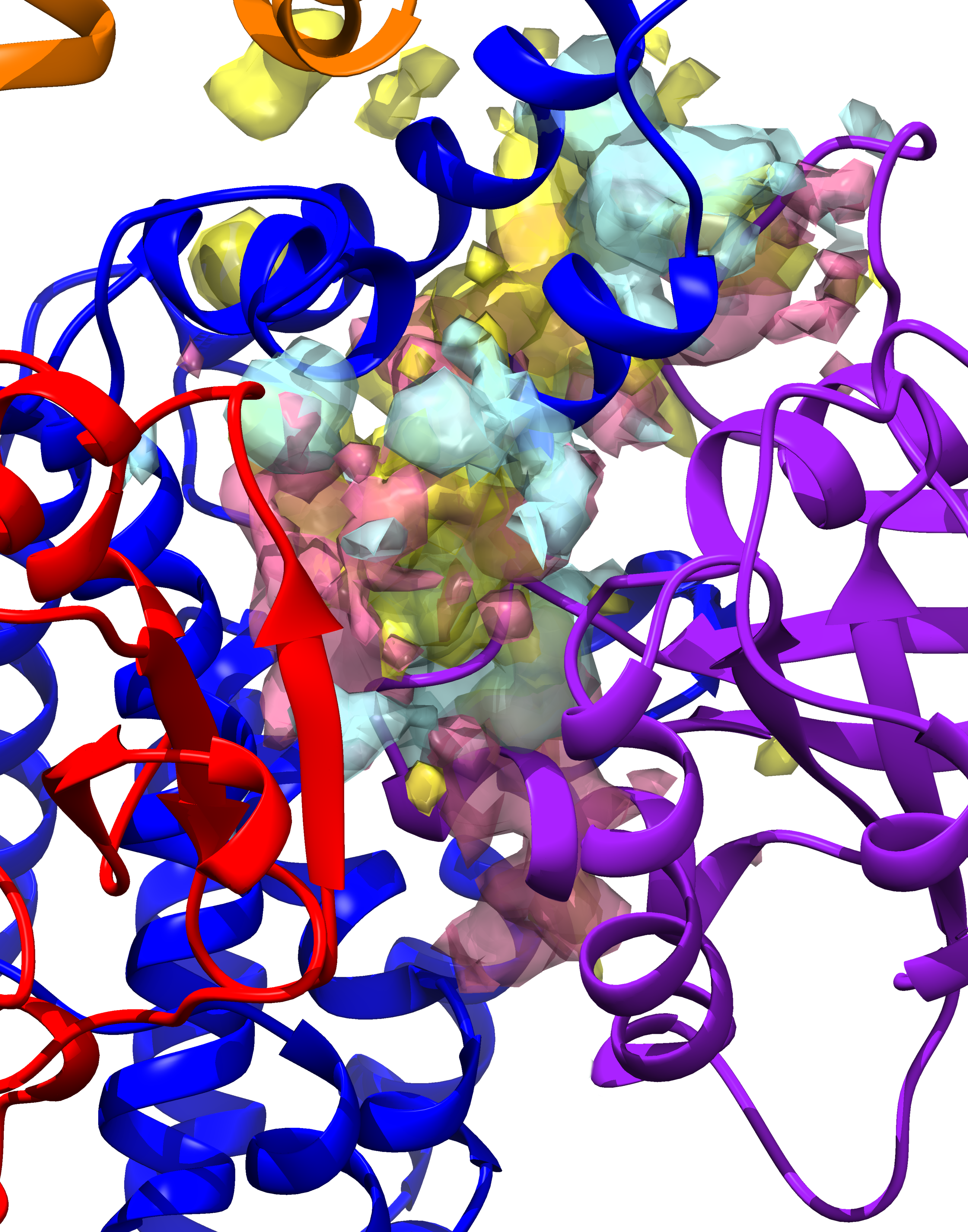  **Area 1** |

Epac1 active conformation with Rap domain. Epac1 is reported as cartoon colored by domains: CNBD and DEP green; REM orange; RA red and CDC25-HD blue. Rap domain is reported as purple cartoon. Yellow maps are for ETA; cyan for ISO and pink for DMSO. cAMP was reported as brown stick

**Fig. S6**

**
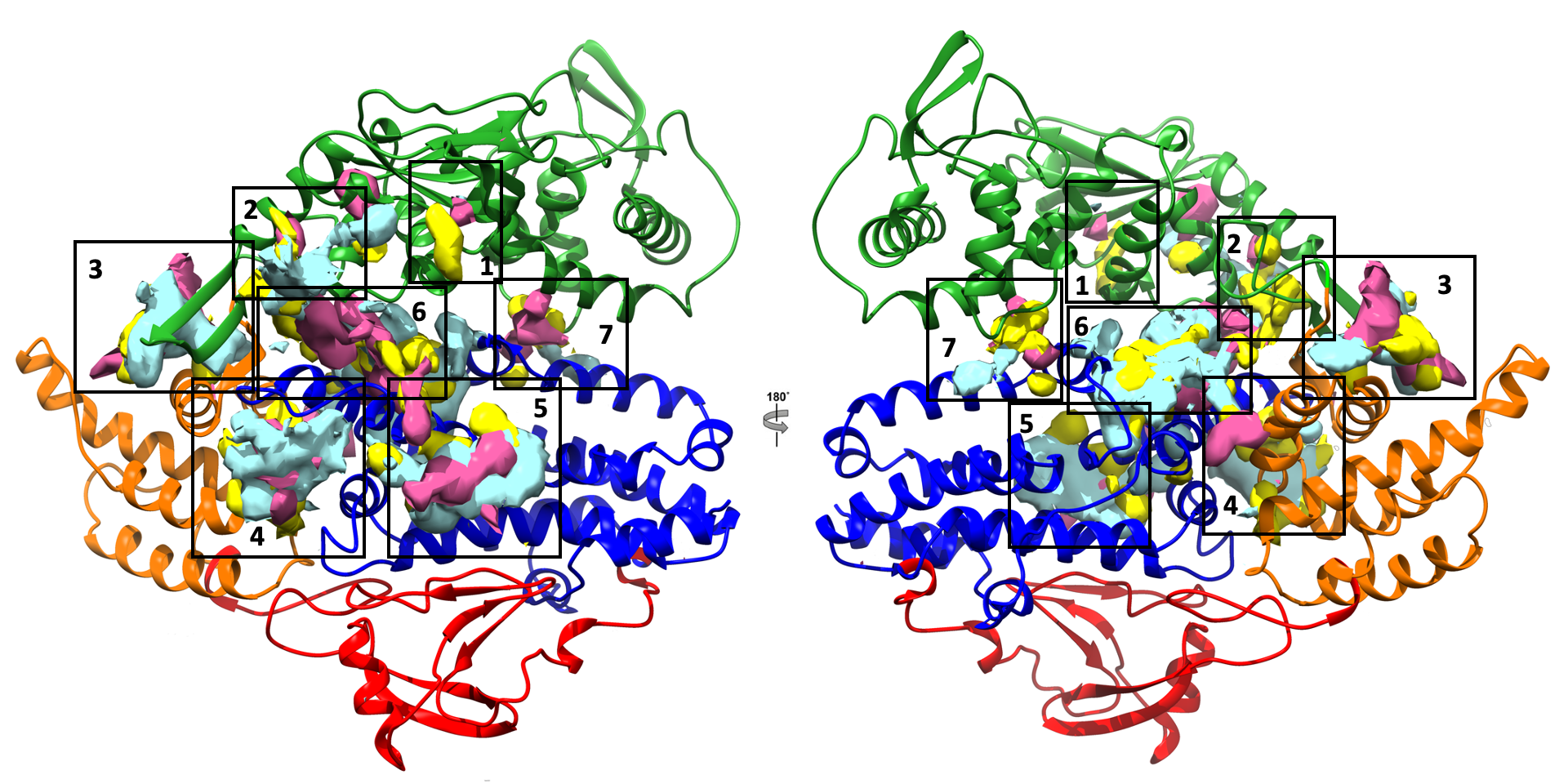
**

Epac1 Csolvents occupancy maps for active conformation by molecurl dynamics of 250ns. Epac1 is reported as cartoon colored by domains: CNBD and DEP green; REM orange; CDC25-HD blue and RA red. Yellow maps are for ETA; cyan for ISO and pink for DMSO. The pictures are rotated of 180 degrees.The areas of cosolvent occupancy maps superimposition are highlighted by black square.

**Fig. S7**


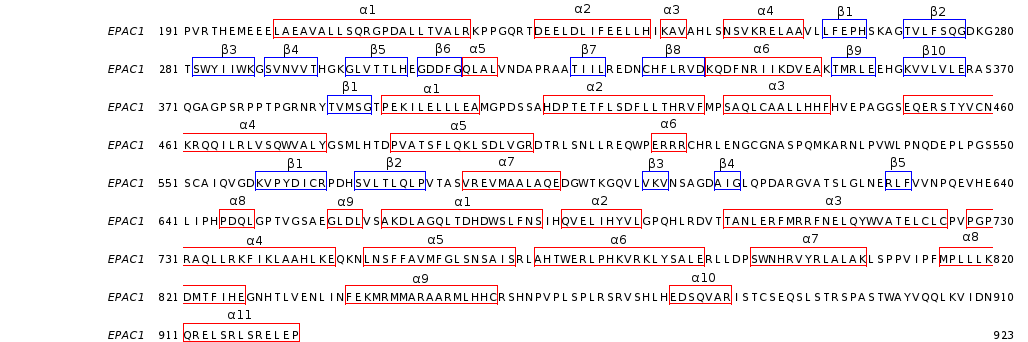


Epac1 sequence and secondary structure. EPAC1 UniProtKB code: O95398. The DEP domain is not included in the sequence secondary assignment. CNBD residues 191-380; REM residues 381-525; RA residues 526-661; CDC25-HD residues 662-923. For each domain the number of the α and β begins from 1.

**Fig. S8**


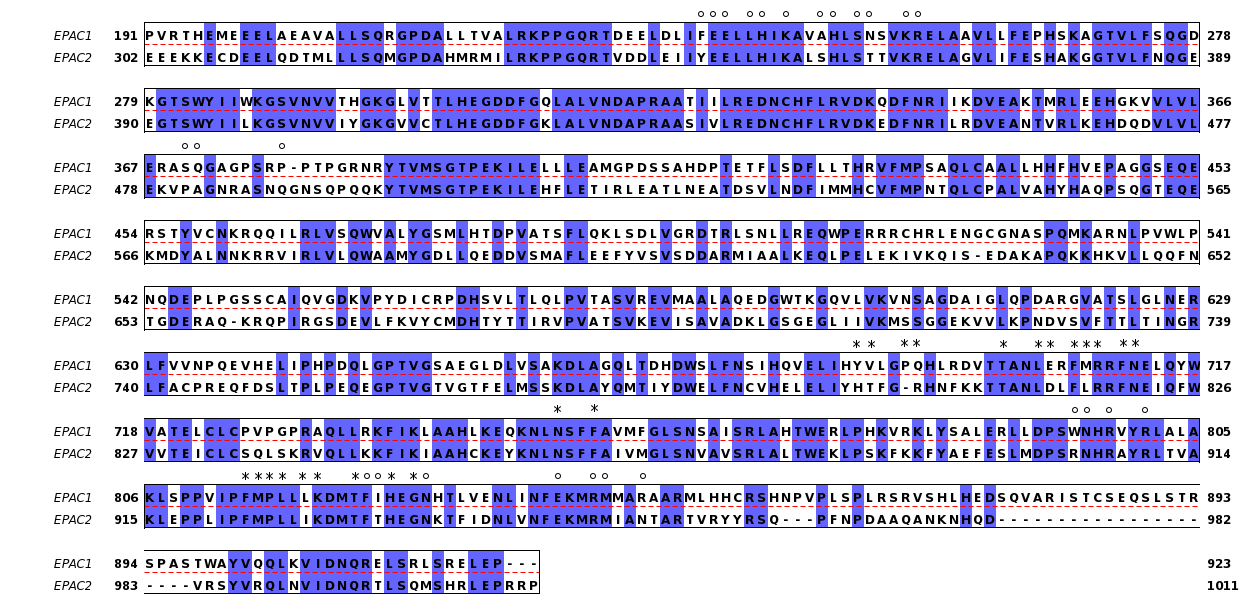


EPAC isoforms sequence alignment and residues of Areas 5 and 6. Epac sequences alignment. Sequences are from uniport, EPAC1 UniProtKB code: O95398, EPAC2 UniProtKB code: Q8WZA2. Conserved residues are highlighted by purple colour. Residues of area 5 are signed by an asterisk. Residues of the area 6 is signed by an empty circle.

**Fig. S9**

| 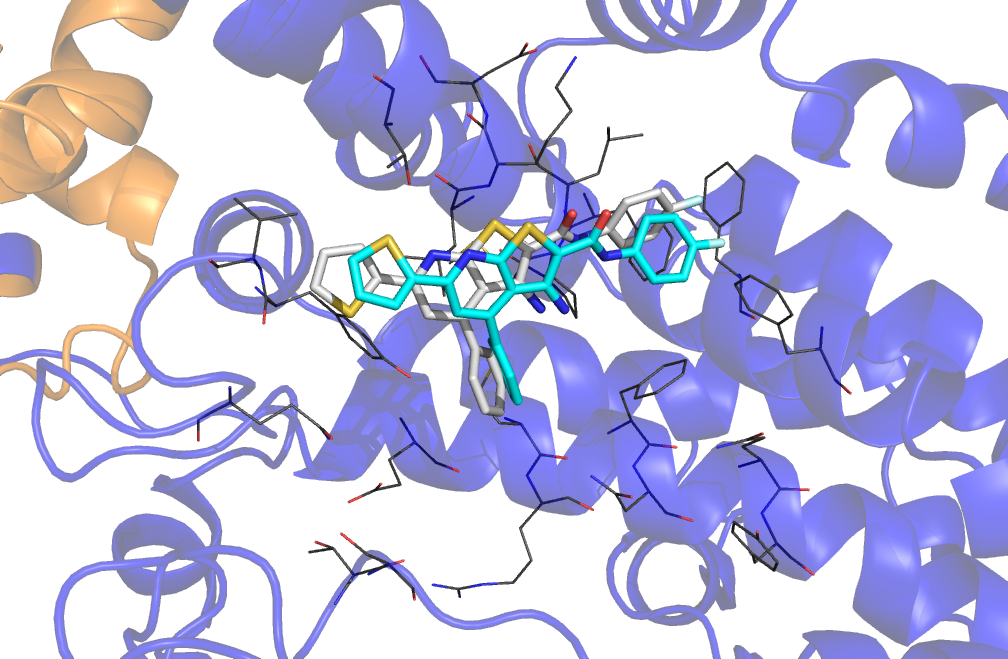 | 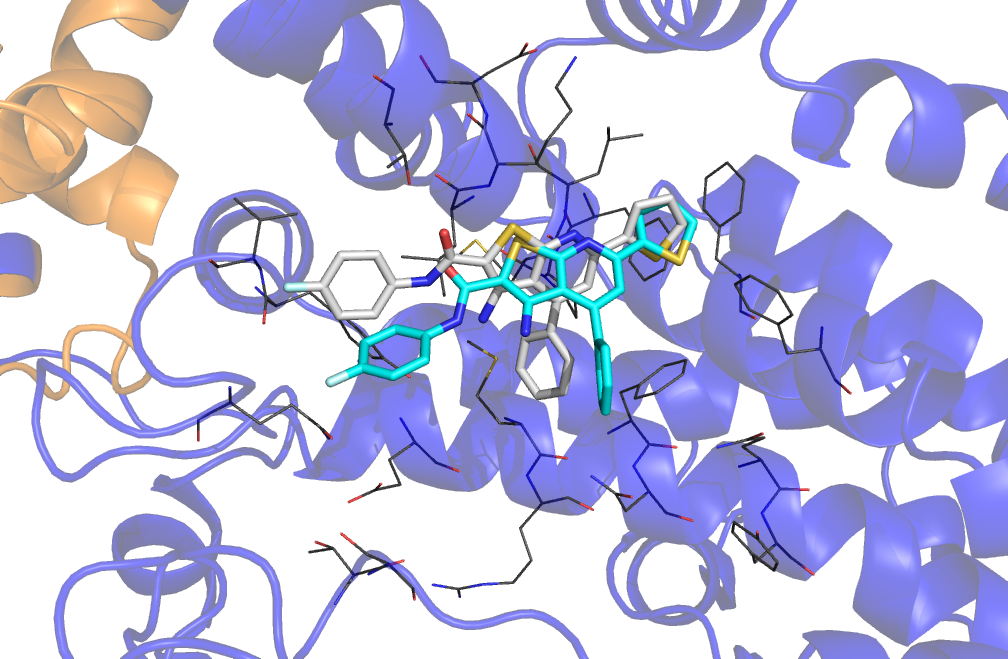 |
| --- | --- |
| Epac1 Area5 pose4_5 and pose9_5 binding modes. Left panel pose4_5; right panel pose9_5. Docking proposed binding mode (white) and molecular dynamic trajectories snapshots (cyan) of AM-001 at Epac1 Area5. The enzyme is reported as cartoon. The domains reported in the picture are, REM orange and CDC25-HD blue. Residues of the pocket are reported as grey lines. | |

**Fig. S10**

| 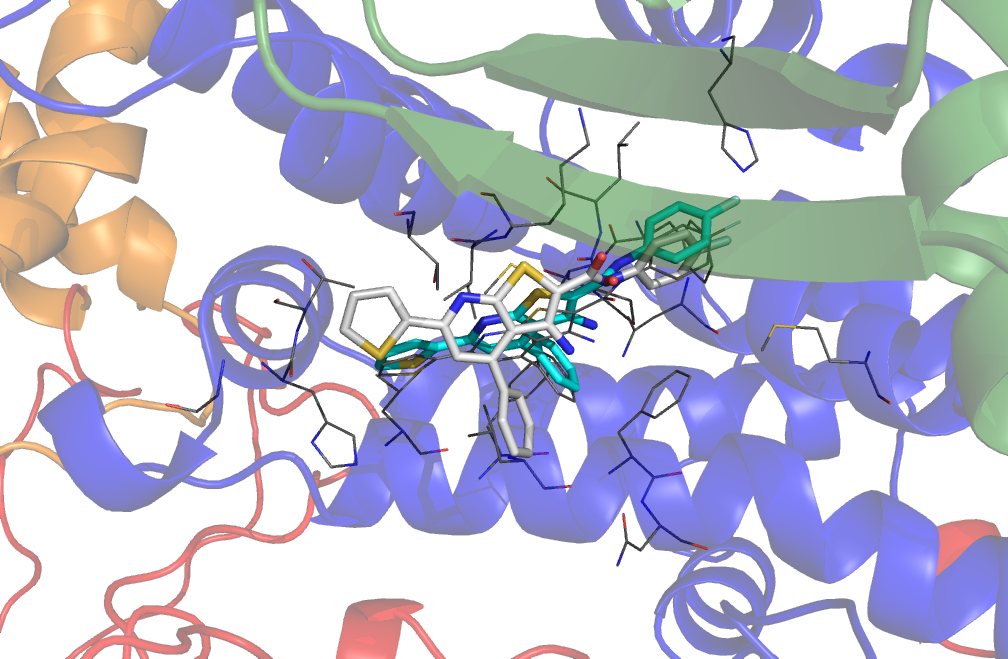 | 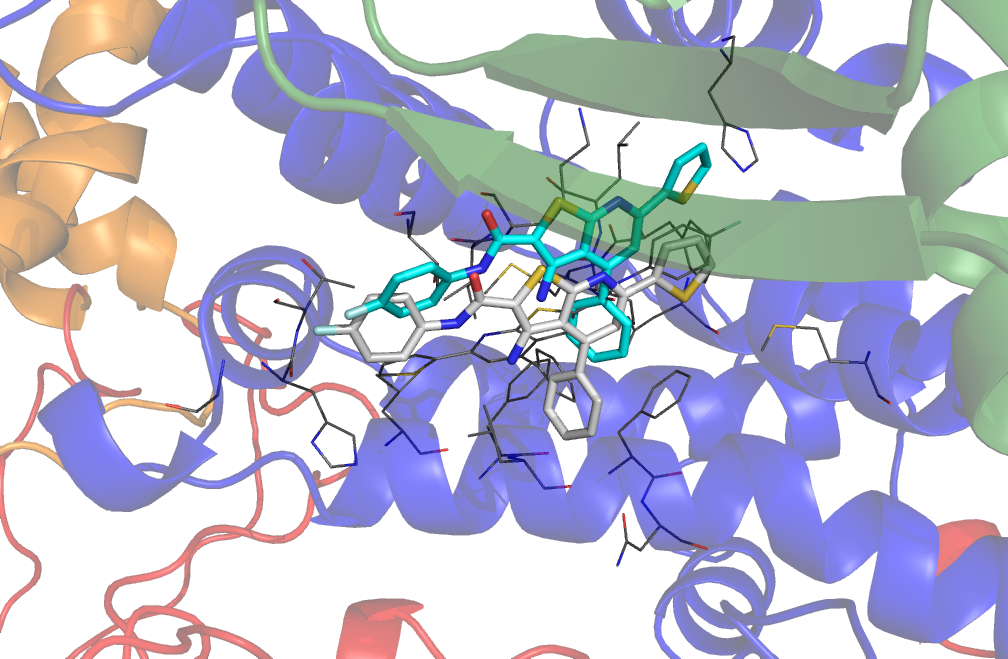 |
| --- | --- |
| Epac2 Area5 pose4_5 and pose9_5 binding mode. Left panel pose4_5; right panel pose9_5. Docking proposed binding mode (white) and molecular dynamic trajectories snapshots (cyan) of AM-001 at Epac2 Area5. The enzyme is reported as cartoon. The domains reported in the picture are, CNBD and DEP green; REM orange; RA red and CDC25-HD blue. Residues of the pocket are reported as grey lines. | |

**Table S1**

| Compounds | Structures | BRET ratio variation^a^ |
| --- | --- | --- |
| AM-001 |  | 46.7 ± 0.8 |
| AM-002 |  | 93.6 ± 0.9 |
| AM-003 |  | 100.4 ± 2.1 |
| AM-004 |  | 47.0 ± 0.7 |
| AM-005 |  | 46.9 ± 1.3 |
| AM-006 |  | 74 ± 2.8 |
| AM-007 |  | 80.5 ± 3.9 |
| AM-008 |  | 82.1 ± 5.5 |
| AM-009 |  | 82.4 ± 1.8 |
| AM-010 |  | 101.3 ± 2.8 |

Structures and Bret ratio variation of AM-001 analogues.^a^

^a^ Data from reference 1

**Fig. S11**

| 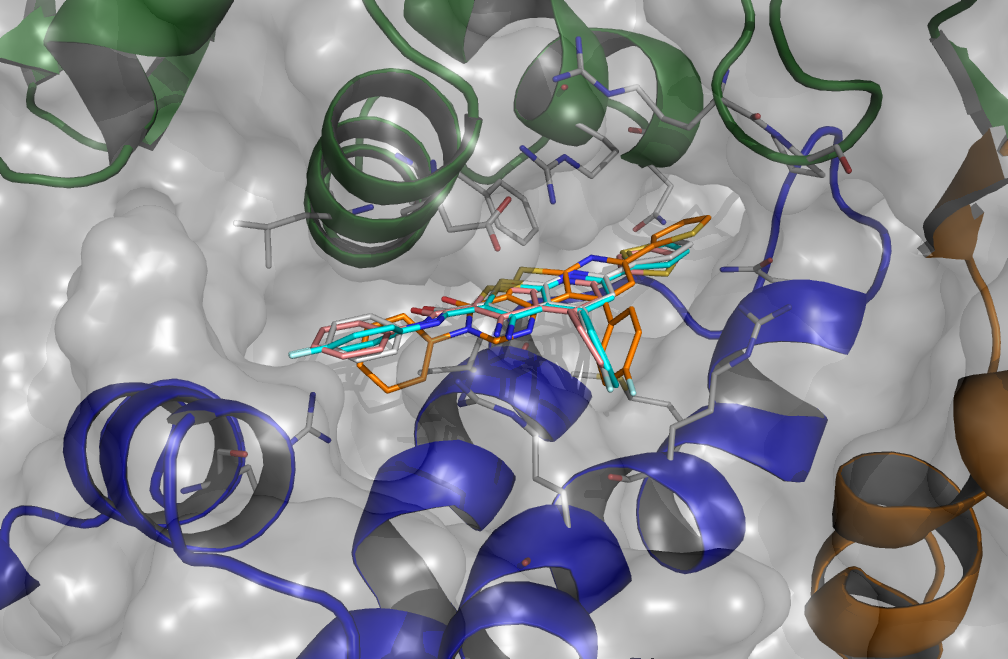 | 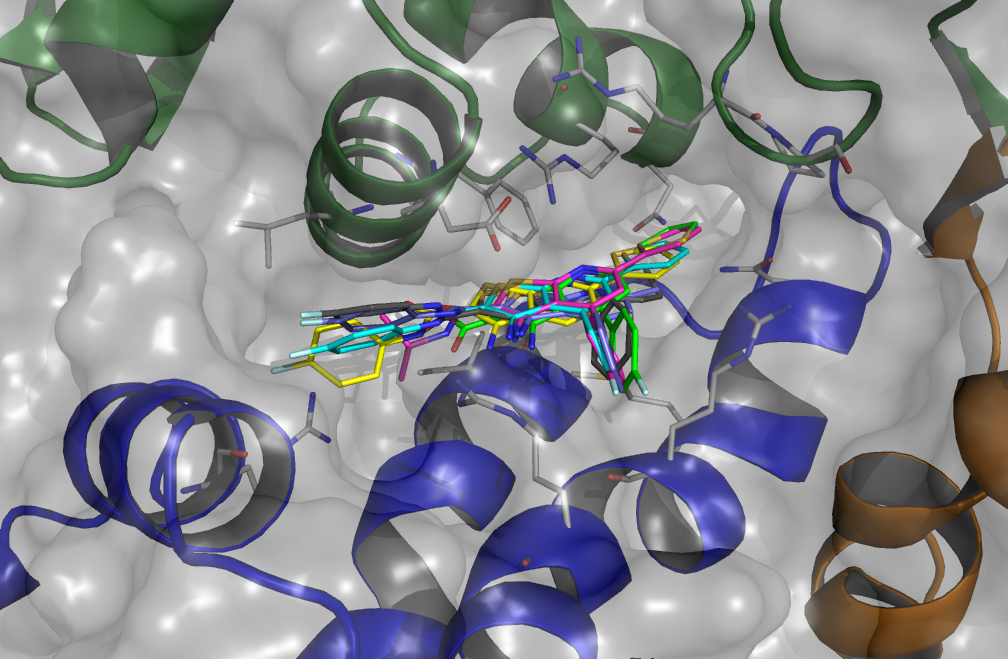 |
| --- | --- |
| Plants proposed binding mode of AM-001 analogues. Left panel: AM-001 (cyan) AM-002 orange, AM-004 pink and AM-005 white; Right panel: AM-001 (cyan), AM-003 magenta, AM-006 light green, AM-007 grey, AM-008 violet and AM-009 yellow. AM-010 is not reported because of unrelated binding mode. Epac1 is reported as cartoon: CNBD and DEP green; REM orange; CDC25-HD blue and RA red. Surface is also reported in grey. Residues involved in interactions were depicted as white stick. | |

**Table S2**

**R = 0.74**

| **Compd** | **ΔG^a^** | **BRET ratio^b^** |
| --- | --- | --- |
| AM-001 | -60.23 | 46.7 |
| AM-002 | -42.65 | 93.6 |
| AM-003 | -49.17 | 100 |
| AM-004 | -49.88 | 47 |
| AM-005 | -58.92 | 46.9 |
| AM-006 | -53.45 | 74 |
| AM-007 | -52.46 | 80.5 |
| AM-008 | -46.1 | 82.1 |
| AM-009 | -47.54 | 82.4 |

Correlation between BRET-ratio values and calculated ΔG (Kcal/mol) of docking proposed binding poses for AM-001 analogues. ^a^ΔG values were calculated by MMGB-SA module of Maestro suite [2, 3]; ^b^data are from reference 1. Compound AM-010 was omitted in the computation by its unrelated binding mode.

The Spearman coefficient Rs was 0.83, p (2-tailed) 0.53%;

The Pearson coefficient R was 0.74

**Fig. S12**

PCs cumulative contribution (%, Y-axis) of variance for the 10 eigenvectors (X-axis) calculated by PCA. The contribution of first and second eigenvectors is also reported to the corresponding point.

| **N° Eigenvectors** | **Proportion of Variance (%)** | | **Eigenvalues** |
| --- | --- | --- | --- |
| 1 | 42.9 | 982.07 | |
| 2 | 15.9 | 363.37 | |
| 3 | 14.1 | 321.60 | |
| 4 | 7.2 | 165.06 | |
| 5 | 5.8 | 133.42 | |
| 6 | 4.4 | 101.89 | |
| 7 | 3.5 | 81.88 | |
| 8 | 3.4 | 77.59 | |
| 9 | 2.6 | 60.75 | |
| 10 | 0.2 | 5.25 | |

**References**

1. Laudette M, Coluccia A, Sainte-Marie Y, Solari A, Fazal L, Sicard P, Silvestri R, Mialet-Perez J, Pons S, Ghaleh B, Blondeau JP, Lezoualc'h F (2019) Identification of a pharmacological inhibitor of Epac1 that protects the heart against acute and chronic models of cardiac stress. Cardiovasc Res 115:1766-1777

2. Jacobson MP, Friesner RA, Xiang Z, Honig B (2002) On the Role of Crystal Packing Forces in Determining Protein Sidechain Conformations, J Mol Biol 320:597-608

3. Schrödinger Release 2018-1: Prime, Schrödinger, LLC, New York, NY, 2018.
